# Free school meals and educational outcomes: evidence from China’s Nutrition Improvement Program

**DOI:** 10.3389/fpubh.2025.1542861

**Published:** 2025-05-22

**Authors:** Zhanli Ma, Yanna Ma, Wei Zou, Zengzeng Fan

**Affiliations:** ^1^School of Statistics and Mathematics, Zhongnan University of Economics and Law, Wuhan, Hubei, China; ^2^Faculty of Applied Economics, University of Chinese Academy of Social Sciences, Beijing, China; ^3^School of Economics and Management, Wuhan University, Wuhan, Hubei, China; ^4^School of Politics and Public Administration, Zhengzhou University, Zhengzhou, Henan, China

**Keywords:** free school meals, educational outcomes, nutritional improvement, family background, educational equality

## Abstract

The education gap between urban and rural areas has long been a significant challenge for developing countries. This study evaluates the impact of China’s large-scale Nutrition Improvement Program (NIP) on the educational outcomes of rural children, investigating the root causes of the urban–rural education divide from the early stages of human capital development. Using data from the China Family Panel Studies (2010–2020), we reveal three notable findings: (1) NIP has significantly boosted rural children’s language grades by 0.223 standard deviations and math grades by 0.172 standard deviations. Over the long term, NIP raised the high school enrollment rate by 8.2%. (2) Heterogeneity analysis indicates that NIP is more effective for relatively disadvantaged groups, such as girls, younger children, and children from low-income families. (3) NIP enhances children’s educational outcomes by improving children’s nutritional intake, strengthening cognitive and non-cognitive abilities, reshaping family educational expectations, and increasing families’ investment in education. Our findings provide valuable insights for refining public policies and contribute significantly to advancing educational equity across regions.

## 1 Introduction

Nutrition improvement is key to enhancing individual health and a crucial foundation for advancing educational achievement, promoting employment, and reducing poverty (1, 2). Proper nutrition is essential for fostering health and economic well-being in low- and middle-income countries. Neglecting nutrition can have lasting negative impacts on a nation and its citizens. Statistics show that malnutrition costs the global economy an estimated $3.5 trillion annually (3). The nutritional challenges in developing countries can be severe, and over the past decade, nutritional improvement has become an increasingly important solution for sustainable development (4).

While education and nutritional health have been strongly correlated, teasing out this causality has been challenging (5, 6). The NIP, a quasi-natural experiment, offers a prime opportunity to explore the causal link between health and education, examining whether such targeted health interventions can enhance educational outcomes. Almost all developed countries have NIP Studies from developed countries such as the United States (7), Sweden (8), United Kingdom (9) have highlighted the positive impact of NIPs on education, including improved scores on language and science tests, higher academic achievement, and extended years of education. However, some studies have reported contrary results. For instance, Gordanier et al. (10) found that the impact of NIP on elementary students’ reading scores in South Carolina was not statistically significant. Bartfeld et al. (11) noted that the NIP in Wisconsin improved standardized reading scores for boys but had no effect on girls. Similar mixed results have been found in developing countries. Research in India and Kenya has indicated that NIPs are correlated with higher test scores (12, 13), indicating that the importance of child health for educational achievement may be greater than previously estimated, considering unobserved factors such as family preferences and health endowments. Due to the short implementation time of China’s NIP policy, few studies have been conducted in China. Fang and Zhu (14) demonstrated that the NIP improved children’s cognitive outcomes. However, some studies have argued that the impact of NIP on education is limited. McEwan (15) evaluated Chile’s NIP, finding no significant effect on fourth graders’ math and language grades. A comparative analysis of school meal programs in developing countries revealed that the positive impact on growth, cognitive abilities, and academic performance of school-age children who received school meals was not as clear as compared to those who did not receive free meals (16).

The main issue with previous research is as follows: Firstly, the impact of NIP on educational outcomes remains uncertain, with a lack of consensus among various studies. Most research has focused on developed countries, where the context of NIP significantly differs from that of developing nations, making it challenging to directly apply these findings to the latter. Secondly, research in developing countries is often limited by small sample sizes and a narrow focus on specific areas or schools. The results of small-scale trials may not align with the effects of large-scale implementation, as the treatment effect may diminish as the scale of implementation rises, meaning that the research conclusions may not serve as an accurate representation of the NIP’s impact. Finally, current research on China’s NIP predominantly centers on children’s health or cognitive abilities, with relatively fewer analyses on educational outcomes. Given the widespread reach of China’s NIP and its impact on a vast population, the question of how to leverage these initiatives to reduce nutrition inequality and, in turn, education inequality is a pressing issue that deserves further study.

No universal consensus on how improved nutrition affects educational achievements has emerged, particularly in the context of China, where studies on the nation’s Nutritional Improvement Program (NIP) have been sparse. China launched the NIP for rural students in the year of 2011, offering free, nutritious meals to improve their health, covering 50 million students in compulsory education, making it one of the world’s most extensive free meal policies (17). Our study focuses on the vulnerable group of rural children to evaluate how improvements in nutrition affect their educational outcomes. We seek to identify and propose policies that can significantly enhance the education of rural children. By making early interventions in the formation of human capital, we hope to bridge the educational divide between urban and rural areas and foster greater educational equity.

Using China’s NIP as a quasi-natural experiment, applying a difference-in-differences (DID) method, we analyze the causal relationship between free school meals and education outcomes. Using microdata from the 2010–2020 China Family Panel Studies (CFPS), we explore the dynamics, intensity, and long-term effects of nutritional improvement on rural children’s educational outcomes. Our findings reveal that NIP has increased language scores by 0.223 standard deviations and math grades by 0.172 standard deviations for children aged 6–15. The NIP has also increased the pass rates for language and math by 6.8 and 5.7%, respectively. Furthermore, the NIP raised high school enrollment in pilot areas by 8.2% for children aged 16–18.

Heterogeneity analysis reveals that the NIP has had a more significant effect on the educational outcomes of younger children, girls, and children from low-education and low-income families, suggesting that the NIP has a significant poverty-reduction effect. However, we also discovered that these positive effects are only evident in areas designated as national pilot areas, and not local areas. Our estimation results indicate that government-subsidized school meal programs positively affect children’s educational outcomes, with more disadvantaged children enjoying greater benefits. Our findings indicate that interventions to improve children’s health during school age are crucial for reducing educational disparities and consequent economic inequalities in adulthood.

The mechanisms by which free school meals improve children’s educational outcomes have primarily focused on the role of nutrition in child physical and mental health. Malnourished children often suffer from severe cognitive delays, and improved nutrition can improve children’s dietary structure, reduce the incidence of malnutrition, and enhance children’s health (18). Research in Ghana indicated that micronutrient-dense foods have a significant influence on NIPs, and nutritious school lunches are associated with higher energy intake and nutritional richness (19). Liu et al. (20) and Bethmann et al. (21) found that nutritious lunches significantly improved students’ psychological health, reducing instances of crying. However, some studies have suggested that nutrition improvement has no significant positive impact on children’s health outcomes, and participants in NIPs did not consume higher-quality diets compared with nonparticipants (22). Wang et al. (23) noted that students participating in NIPs generally have better health. A survey of 100 rural primary schools in northwest China found that the NIP did not reduce the incidence of malnutrition among the sampled students (24).

NIP affects children’s educational outcomes through multiple mechanisms in our study. Regarding the nutritional input mechanism, these initiatives increase children’s consumption of fish, eggs, milk, fruit, and vegetables, enhancing their dietary intake. From the capability perspective, educational outcomes are a composite reflection of children’s abilities, and the NIP partially boosts children’s cognitive performance, in terms of noncognitive skills and heightens their effort, self-discipline, and concentration in learning, in addition to instilling a sense of responsibility among children. Examining the family education mechanism, the NIP has also raised family educational expectations and altered consumption patterns to allow families to allocate more resources to children’s educational investments.

The innovative aspects of this article are reflected in the following key marginal contributions: Firstly, the study broadens the research horizon on the impact of nutritional improvement on educational outcomes, particularly in the context of rural China. Existing studies have predominantly focused on the effects of the Nutritional Improvement Program (NIP) on the cognitive abilities of children aged 10 to 16, often neglecting the younger age group (25). Additionally, studies have typically concentrated on the effects of NIP within a specific year, failing to account for the NIP’s continuity and long-term impact, which has led to inconsistent research findings (26). Secondly, we provide a nuanced analysis of the NIP’s target demographics, encompassing children of different ages and family backgrounds, to determine whether the NIP improved vulnerable rural children’s educational outcomes and fulfilled the policy’s intended goals. We analyze various aspects of the NIP at different levels, assessing the anticipated effectiveness of funding, broadening policy understanding of China’s NIP, and systematically examining its significance. The comprehensive policy assessment also offers valuable insights for developing similar initiatives in other developing countries. Lastly, the study reveals the mechanisms by which the NIP affects educational performance, providing a clear path for future enhancements to the program and offering insights into effective policies that could help bridge the education divide between urban and rural areas and foster greater educational equity.

The remainder of this paper is structured as follows. Section 2 introduces the policy background of China’s NIP, Section 3 presents the data and methodology of our empirical analysis, Section 4 details the results and heterogeneity analysis, Section 5 discusses the mechanism analysis, and finally, Section 6 concludes.

## 2 Policy background

The nutritional challenges in developing countries are severe. The United Nations’ 2030 Agenda for Sustainable Development includes the global goal of eliminating all forms of malnutrition by 2030. In 2022, 34.7% of children faced various forms of malnutrition, with the rates of stunting and wasting in rural areas being 1.6 times and 1.4 times higher than in urban areas globally (27). To accelerate the development of rural education and promote educational equity, in November 2011, China launched the NIP for rural students in compulsory education, providing free nutritious lunches. Before 2011, while various local and charity-driven efforts aimed to address malnutrition among rural students, this was the first nationwide policy to directly tackle the issue on such a large scale. Initially, central finance provided nutritional meal subsidies for rural students in 699 poverty-stricken counties, and by the end of 2022, the number of national plan counties reached 726. In addition to specifying meal standards, the policy also allocated funds to improve dining conditions, build and renovate school canteens, and vigorously promote school canteen meal services. Since NIP’s implementation, the nutritional status of rural children in China has improved considerably. The program has seen a gradual increase in the dietary standards for free nutritious lunches, from 3 yuan per student per day in 2011 to 5 yuan per student per day in 2021. By the end of 2021, the central government had allocated a total of 196.734 billion yuan for student nutrition and dietary subsidies. According to the Report on Nutrition and Chronic Disease Status of Chinese Residents 2015, the malnutrition rate among rural children and adolescents aged 6–17 was 4.7% in 2012 and dropped to 2.2% in 2015 (28).

In terms of implementation bodies, in addition to the national NIP, various provinces and cities have also implemented targeted NIPs based on local conditions. National and provincial local NIPs differ in implementation standards, methods, funding sources, and supervision. In terms of implementation intensity, the subsidy standards for China’s NIP have varied at different times. The meal standards for the free nutritious lunch have been gradually expanding. In 2015, the subsidy standard for the NIP was raised from 3 yuan per student per day to 4 yuan. The Chinese government has invested substantial resources, including human, material, and financial capital, to advance the NIP. Consequently, a detailed and accurate assessment is needed to determine how the NIP has impacted the educational outcomes of rural children and whether it has achieved the initial intent of the policy. NIP is rich and diverse in China, providing conditions for a detailed assessment of the policy’s effects.

## 3 Data and research design

### 3.1 Data

We use data from the CFPS, a far-reaching social tracking survey that captures data across individual, family, and community levels and offers a comprehensive view of the evolution of China’s society, economy, population dynamics, educational landscape, and health trends. The CFPS is a large-scale, nationwide survey that includes a representative sample of the population from 25 provinces, municipalities, and autonomous regions. Conducted biennially from 2010 to 2020, the survey encompasses newly added families and follow-up visits with previous participants, with all baseline family members from 2010 marked for ongoing observation. The child questionnaire within the CFPS examines children’s attributes, academic achievements, dietary habits, cognitive skills, and non-cognitive abilities, which are highly relevant to the scope of our study.

We examine the impact of free school meals on the educational outcomes of rural children. The samples are restricted to children aged 6 to 15 with rural household registration, enrolled in primary or junior high school, and exclude samples that did not identify the county, age, or household registration. Given the heterogeneous implementation standards and varying degrees of enforcement and feedback in local pilot areas, our baseline regression analysis only considers national pilot areas.

### 3.2 Identification strategy

Due to variations in policy implementation timing and regional disparities, this study employs the DID approach to evaluate the impact of the NIP on rural children’s educational outcomes. The baseline regression estimates are shown in Equation (1):


(1) $$y_{\mathrm{ict}}=\alpha +{\text{βtreat}}_{c}\times {\text{post}}_{t}+X\prime \gamma +\theta_{c}+\tau_{t}+\pi_{i}+\varepsilon_{\mathrm{ict}}$$

where subscripts i , c , and t represent individuals, districts, and years, respectively. $y_{\mathrm{ict}}$ captures rural children’s educational outcomes, serving as our dependent variable. treat is a dummy variable indicating whether a district is a pilot area. If it is a pilot area, it is assigned a value of 1, otherwise 0. post denotes policy implementation time; when the time is after the policy was implemented, it is assigned a value of 1, otherwise 0. ${\text{treat}}_{c}\times {\text{post}}_{t}$ is a binary variable indicating whether county c in year t was affected by the policy, which is coded as 1 if affected and 0 otherwise. The coefficient β is the value of primary interest in this study, which allows us to approximate the Average Treatment Effect (ATE) of the NIP on grades of students. X′ denotes a series of control variables. To control for individual characteristics, district/county characteristics, and time characteristics that do not change over time, we introduce individual fixed effects ( $\pi_{i}$ ), county fixed effects ( $\theta_{c}$ ), and time fixed effects ( $\tau_{t}$ ) into the estimation. The reason for adding district/county fixed effects is that population mobility occurred during the survey period, and some children have migrated, with inconsistencies between the samples’ household registration and place of residence; therefore, individuals and districts/counties do not completely correspond. $\varepsilon_{\mathrm{ict}}$ is the error term. The standard error of this model is clustered at the county level. The NIP was initiated in autumn 2011 and spring 2012 in our data, we determine the timing of the policy implementation by combining the year and month of the survey in different counties.

### 3.3 Variable descriptions and descriptive statistics

The central focus of this study is children’s educational outcomes, which we measure by language and math grades. The data are obtained from the CFPS parent survey question, “To your knowledge, how is the child’s academic performance?” with responses categorized as excellent, good, average, or poor, which we translate into numerical values ranging from 4 to 1, respectively. We also standardize the grades for language and mathematics, with higher scores reflecting better academic performance. Beyond these metrics, other variables of interest include the pass rates for Chinese and mathematics and high school enrollment rates.

The model incorporates control variables across dimensions of individual child characteristics, parental attributes, and district-level features. Individual child characteristics encompass the child’s sex and age, while parental attributes include the ages and educational attainment of both parents. At the district level, we control for variables of per capita GDP, which is in the logarithmic form, the share of the primary sector in the economy, the secondary sector’s proportion, the student–teacher ratio in primary schools, and the proportion of local educational spending relative to total fiscal expenditure.

Table 1 presents the descriptive statistics of our dataset, which includes 106 counties, 21 of which are national pilot areas and 85 nonpilot areas after excluding local pilot areas. The dataset contains 1,587 child samples and 7,044 observations, with 2,045 observations from pilot areas and 4,999 from nonpilot areas.

**Table 1:** Descriptive statistics.

Variables	Observations	Mean	Standard deviation	Min	Max
Language grades	7,044	0	1	−1.771	1.314
Math grades	7,044	0	1	−1.639	1.272
NIP	7,044	0.172	0.378	0.000	1.000
Gender	7,044	0.531	0.499	0.000	1.000
Age	7,044	11.178	2.474	6.000	15.000
Father’s highest years of education	7,044	7.721	2.809	0.000	16.000
Mother’s highest years of education	7,044	6.652	2.82	0.000	16.000
Father’s age	7,044	39.755	5.641	25.000	65.000
Mother’s age	7,044	37.874	5.512	22.000	74.000
Per capita GDP	7,044	10.660	0.566	8.736	12.480
Primary industry share	7,044	0.065	0.051	0.001	0.234
Secondary industry share	7,044	0.466	0.114	0.095	0.854
Primary school teacher–student ratio	7,044	0.18.212	0.03.741	0.072	0.293
Education expenditure share	7,044	0.173	0.052	0.017	1.667

## 4 Empirical results and heterogeneity analysis

### 4.1 Baseline regression results

This study conducts analyses based on three samples: Firstly, the baseline regression is shown in columns (1) and (2) of Table 2. Secondly, although regional control variables are included in the model, considering the significant differences in economic development levels and educational resources across cities, samples from districts and counties in large cities such as Beijing and Shanghai are excluded. The regression results are shown in columns (3) and (4) of Table 2. Finally, considering the existing inconsistencies between household registration and birthplace among the floating population, we exclude floating population samples and rerun the estimation, as shown in columns (5) and (6) of Table 2. From the benchmark regression, the NIP has improved rural children’s language grades by 0.223 standard deviations and math grades by 0.172 standard deviations. The results are also significant after excluding the samples of provincial capitals and municipalities directly under the central government and floating population samples. This indicates that the NIP improves rural children’s education outcomes and has a positive impact on rural children’s accumulation of human capital. This narrows the gap in human capital between urban and rural areas and promotes educational equality.

**Table 2:** Analysis of the impact of the NIP on children’s educational outcomes.

Variable	Benchmark regression		Drop provincial capitals and direct-controlled municipalities		Drop the floating population	
	(1)	(2)	(3)	(4)	(5)	(6)
	Language	Math	Language	Math	Language	Math
Policy effect	0.223***	0.172*	0.221***	0.186*	0.199***	0.183**
	(0.084)	(0.096)	(0.073)	(0.100)	(0.072)	(0.081)
Control variables	Yes	Yes	Yes	Yes	Yes	Yes
District fixed effects	Yes	Yes	Yes	Yes	Yes	Yes
Year fixed effects	Yes	Yes	Yes	Yes	Yes	Yes
Individual fixed effects	Yes	Yes	Yes	Yes	Yes	Yes
Observations	7,044	7,044	6,848	6,848	6,733	6,733
R-squared	0.561	0.594	0.563	0.596	0.568	0.601

***, **, and * represent significance at 1, 5, and 10% levels, respectively, and robust standard errors are in parentheses.

### 4.2 Parallel trend test: event study

The benchmark regression reveals that the NIP has had a positive impact on rural children’s education outcomes. We further estimate the dynamic effect of the educational outcomes over time using the event study method (29), constructing interaction terms between the experimental group binary variable and each year, which can also test the validity of the benchmark regression. The regression estimates are shown in Equation (2):


(2) $$y_{\mathrm{ict}}=\alpha +\sum_{t=2010}^{2020}\varphi_{t}{\text{treat}}_{c}\times {\text{year}}_{t}+X\prime \gamma +\theta_{c}+\tau_{t}+\pi_{i}+\varepsilon_{\mathrm{ict}}$$

**Figure 1:**
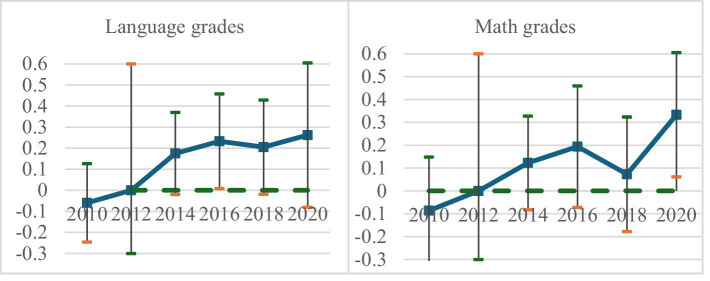
Dynamic effect analysis. The horizontal axis represents the year, the left graph’s vertical axis represents the estimated coefficient of the language grade, and the right graph’s vertical axis represents the estimated coefficient of the math grade. The vertical solid lines in the graph represent the 95% confidence interval.

Where year is the dummy variable for the corresponding year, which is assigned a value of 1 in ${\text{year}}_{t}$ and otherwise 0. Since the CFPS collects data every 2 years, we take 2012 as the reference point to monitor annual fluctuations in coefficients before and after the policy’s rollout. Figure 1 illustrates the mean differences in language and math grades among children in national pilot regions, comparing these to other counties during the baseline period before and after NIP implementation. Initially, the grades in pilot areas were marginally lower than those in non-pilot areas, although insignificant. However, following the introduction of the NIP, the disparity between pilot and nonpilot areas began to narrow, with coefficient values moving from negative to positive territory. For example, before the policy, language grades in pilot areas lagged behind nonpilot areas by 0.32, but this gap narrowed over time. By 2020, language grades in pilot counties had risen by 0.028 relative to non-pilot counties. The trend for mathematics scores also exhibited a generally upward trajectory. The dynamic effects suggest that the coefficients exhibit an increasing trend post policy implementation, indicating a strengthening of the policy’s impact over time and highlighting the long-term benefits of the NIP. The event study approach further validates that our study adheres to the parallel trend assumption, confirming that the baseline regression provides reliable estimates.

### 4.3 Robustness test: placebo test

**Figure 2:**
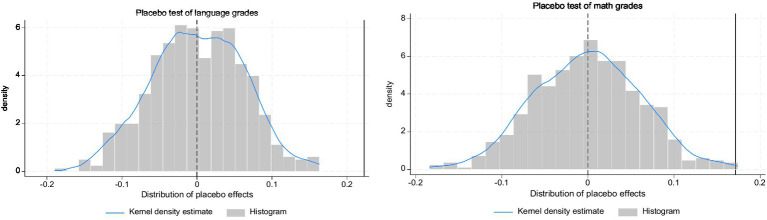
Kernel density plot and histogram of the placebo test.

A key presupposition of the DID methodology is the parallel trend assumption, which posits that the outcomes for treatment and control groups would follow similar trajectories without treatment. However, once the treatment is applied, disentangling the treatment’s impact from broader temporal trends becomes challenging, rendering the parallel trend assumption inherently unverifiable. To address this concern, we conducted placebo tests to explore the presence of any overlooked confounding events that could skew our results. Our analysis employs an individual placebo test, maintaining the treatment timing and the structure of county groupings while randomly reassigning individuals to different counties. We then conduct two-way fixed effects modeling and repeat this 500 times to generate a kernel density plot and histogram of the placebo effects. The visualizations in Figure 2 reveal that the majority of data points cluster around zero on the horizontal axis. The estimated treatment effect (denoted by a vertical solid line in the graph) is situated in the right tail of the placebo effect distribution, suggesting that it is an outlier. This positioning supports the robustness of our DID model results, indicating that our findings are not likely due to chance fluctuations but to the actual impact of the intervention.

### 4.4 Intensity effect

The subsidy standards for the NIP are dynamically adjusted. Within our observation period, a notable change occurred in 2015 when China’s central fiscal policy raised the subsidy standard for the national NIP pilot areas from an average of 3 yuan to 4 yuan per student school meal. To assess the efficiency of the use of fiscal funds, we formulated the following to evaluate the policy’s intensity effect we formulated Equation (3) to evaluate the policy’s intensity effects:


(3) $$y_{\mathrm{ict}}=\alpha +{\text{βtreat}}_{c}\times {\text{post}}_{t}\times {\text{subsidy}}_{t}+X\prime \gamma +\theta_{c}+\tau_{t}+\pi_{i}+\varepsilon_{\mathrm{ict}}$$

where subsidy refers to the fiscal subsidy standard in year t . We introduce an interaction term between the subsidy amount, which serves as a measure of policy intensity, and pilot areas to reflect the impact of each one yuan increase in the standard of free school meals on children’s educational outcomes. As shown in Table 3, this increase corresponds with a 0.062 improvement in the standard deviation of rural children’s language grades and a 0.058 standard deviation improvement in math grades. The enhancement of the school meal standard exhibits an upward trend in its beneficial effect on children’s educational achievements.

**Table 3:** Intensity effect of the NIP.

Variable	Benchmark regression		Exclude provincial capitals and direct-controlled municipalities		Exclude the floating population	
	(1)	(2)	(3)	(4)	(5)	(6)
	Language	Math	Language	Math	Language	Math
Intensity effect	0.062**	0.058*	0.060**	0.063*	0.056**	0.070***
	(0.026)	(0.031)	(0.026)	(0.032)	(0.024)	(0.026)
Control variables	Yes	Yes	Yes	Yes	Yes	Yes
District fixed effects	Yes	Yes	Yes	Yes	Yes	Yes
Year fixed effects	Yes	Yes	Yes	Yes	Yes	Yes
Individual fixed effects	Yes	Yes	Yes	Yes	Yes	Yes
Observations	7,044	7,044	6,848	6,848	6,733	6,733
R-squared	0.560	0.594	0.563	0.596	0.567	0.600

***, **, and * represent significance at 1, 5, and 10% levels, respectively, and robust standard errors are in parentheses.

### 4.5 Long-term effects

This baseline analysis is based on parental reports concerning the selection of educational performance variables. Although the survey questionnaire asked parents to select their children’s academic performance in the previous semester, it may be difficult to avoid subjectivity and potential bias. We have also modified the indicators to consider the long-term effects of NIP on educational outcomes. We adopt two methods. First, from the marginal perspective, we consider the impact of the NIP on children with relatively weak academic performance. We divide children’s grades into “excellent, good, average” as passing, assigning a value of 1, while “poor” is considered as failing and assigned a value of 0. The regression results are presented in Table 4. Columns (1) and (2) show that the NIP significantly increased the passing rates of rural children’s language and math grades by 5%, with the language passing rate increasing by 5.7% and the math passing rate increasing by 6.8%. This indicates that the NIP has a promotional effect on children with relatively poor academic performance and increases the possibility of receiving further education.

**Table 4:** Long-term effect analysis of the NIP.

Variable	Pass rate		High school enrollment rate	
	(1)	(2)	(3)	(4)
	Language	Math	High school enrollment	High school enrollment
Policy effect	0.057**	0.068**		
	(0.028)	(0.031)		
Policy effect 1			0.082***	
			(0.027)	
Policy effect 2				0.031***
				(0.007)
Control variables	Yes	Yes	Yes	Yes
District fixed effects	Yes	Yes	Yes	Yes
Year fixed effects	Yes	Yes	Yes	Yes
Individual fixed effects	Yes	Yes	Yes	Yes
Observations	7,044	7,044	4,053	4,053
R-squared	0.496	0.509	0.341	0.343

***, **, and * represent significance at 1, 5, and 10% levels, respectively, and robust standard errors are in parentheses.

Second, we replace language and math grades with the high school enrollment rate to examine the long-term effect of the NIP on children’s educational outcomes. We selected individuals who were 16–18 years of age during the survey period. If their highest education level was high school or above, including those studying in high school, they were assigned a value of 1, and otherwise 0.

To assess the long-term impact of the NIP on individual high school admissions, this study considers two methods. First, we constructed a variable based on whether a student was affected by the NIP. For the second method, we construct a variable based on the number of years a student benefited from the NIP and change the NIP measurement method. The results are presented in Table 4, columns (3) and (4). Column (3) demonstrates that the NIP increased the high school enrollment rate of children in pilot areas by 8.2%. Column (4) is significant at the 1% level, indicating that for each additional year of benefiting from the NIP during the compulsory education stage, students’ high school enrollment rate increases by about 3.1%. This study evaluates the NIP from the perspective of long-term effects, revealing the long-term positive impacts of the NIP on improving rural educational outcomes.

This section examines the baseline effect of the NIP on educational outcomes, while also considering the NIP’s dynamic effects, long-term effects, and intensity effects. These analyses confirm the significant improvement in educational outcomes in pilot areas due to the NIP, thereby demonstrating the reliability of the conclusions drawn in this paper.

### 4.6 Heterogeneity analysis

#### 4.6.1 Heterogeneity analysis based on family background

This study next conducts a heterogeneity analysis, examining how the NIP affects children from different family backgrounds. We analyze whether the NIP has improved the educational outcomes of children from relatively disadvantaged families. We measure the educational background of the family by the highest level of education of both parents, which we divide into high and low categories based on the boundary of compulsory education. We also categorize families based on income status, considering those with annual net income in the bottom 30% as low-income families and the rest as middle-to-high-income families, referencing the criteria of Gustafsson and Sai (30). This classification enables us to assess the impact of the NIP on families with varying income levels.

The results are presented in Table 5. The estimation results demonstrate that the NIP has no significant effect on children from families with high educational or middle-to-high-income backgrounds, but the NIP promotes the children’s accumulation of human capital from low-income and low-educational families, with a greater effect on improving the next generation’s human capital in relatively poor families. Poverty alleviation must start with education, and the NIP is conducive to preventing the intergenerational transmission of poverty and promoting low-income families’ upward mobility.

**Table 5:** Policy effect evaluation for families with different backgrounds.

Variable	High		Low	
	Language	Math	Language	Math
policy effect for families with different educational backgrounds				
Policy effect	− 0.020	0.110	0.249***	0.175*
	(0.226)	(0.233)	(0.089)	(0.098)
Observations	896	896	6,148	6,148
R-squared	0.615	0.622	0.561	0.595
policy effect for families with different income backgrounds				
Policy effect	0.204	0.135	0.199	0.317**
	(0.136)	(0.137)	(0.144)	(0.128)
Observations	4,492	4,492	2,138	2,131
R-squared	0.637	0.666	0.749	0.757
Control variables	Yes	Yes	Yes	Yes
District fixed effects	Yes	Yes	Yes	Yes
Year fixed effects	Yes	Yes	Yes	Yes
Individual fixed effects	Yes	Yes	Yes	Yes

***, **, and * represent significance at 1, 5, and 10% levels, respectively, and robust standard errors are in parentheses.

#### 4.6.2 Heterogeneity analysis based on individual gender

In rural China, particularly in impoverished regions, the traditional preference for sons over daughters remains prevalent, often leading to the neglect of girls’ nutritional well-being. To address this, we further examined the differential responses and outcomes of boys and girls to NIP. The results are presented in Table 6. Columns (1) and (2) show that the NIP has a statistically significant positive impact on language grades for boys. Columns (3) and (4) show that the NIP has a significant positive impact on both language and math grades for girls, who are a relatively vulnerable group. The result revealed that such programs had a more pronounced impact on the educational achievements of girls. By compensating for the lack of nutritional investment in girls within their families, these initiatives help unlock their academic potential, thereby contributing to the reduction of gender inequality in rural areas during adolescence.

**Table 6:** Policy effect evaluation for different genders.

Variable	Male		Female	
	(1)	(2)	(3)	(4)
	Language	Math	Language	Math
Policy effect	0.234**	0.075	0.226**	0.293***
	(0.108)	(0.122)	(0.113)	(0.111)
Control variables	Yes	Yes	Yes	Yes
District fixed effects	Yes	Yes	Yes	Yes
Year fixed effects	Yes	Yes	Yes	Yes
Individual fixed effects	Yes	Yes	Yes	Yes
Observations	3,743	3,743	3,301	3,301
R-squared	0.559	0.613	0.553	0.584

***, **, and * represent significance at 1, 5, and 10% levels, respectively, and robust standard errors are in parentheses.

#### 4.6.3 Heterogeneity analysis based on different individual ages

We examine the impact of the NIP on children of different ages (Table 7). Children aged 6–15 are in a critical period of physical development, and those in different age stages require different amounts of fat, protein, carbohydrates, and main trace elements (31). Therefore, we divide the children into three age stages encompassing 6–9 years old, 9–12 years old, and 13–15 years old to assess the effect of the NIP. Table 7 reveals that the NIP is significant for the language and math grades of younger children, and from the size of the coefficients, the grade improvement is the largest, with the best policy effect. While language grades are significant for children aged 10–12, the impact on children participating in the NIP aged 13–15 is insignificant. This indicates that earlier policy intervention has better effects, and the implementation effect is better for younger than older children. Early policy intervention can effectively improve the efficiency of fiscal fund use and achieve expected positive results.

**Table 7:** Policy effect evaluation for different age groups.

Variable	Ages 6–9		Ages 10–12		Ages 13–15	
	(1)	(2)	(3)	(4)	(5)	(6)
	Language	Math	Language	Math	Language	Math
Policy effect	0.250**	0.234**	0.219**	0.097	0.051	−0.063
	(0.098)	(0.106)	(0.095)	(0.104)	(0.262)	(0.226)
Control variables	Yes	Yes	Yes	Yes	Yes	Yes
District fixed effects	Yes	Yes	Yes	Yes	Yes	Yes
Year fixed effects	Yes	Yes	Yes	Yes	Yes	Yes
Individual fixed effects	Yes	Yes	Yes	Yes	Yes	Yes
Observations	4,281	4,281	5,808	5,808	3,292	3,292
R-squared	0.530	0.556	0.615	0.652	0.774	0.806

***, **, and * represent significance at 1, 5, and 10% levels, respectively, and robust standard errors are in parentheses.

#### 4.6.4 Heterogeneity analysis based on boarding circumstances

Low-age boarding is a major issue faced by rural compulsory education and is also a difficulty and challenge faced by national compulsory education in China. By the end of 2017, 9.346 million boarding students attended rural primary schools, accounting for 14.1% of the total number of rural primary school students (32). This study examines children who indicate boarding or non-boarding status to explore the impacts of the NIP on the educational outcomes of children with different boarding situations. The results are presented in Table 8, revealing that the NIP significantly improves boarding students’ language grades, with a superior impact on the educational outcomes of boarding students than non-boarding students. In summary, our heterogeneity analysis at the individual and family levels demonstrates that NIP implementation has significantly improved the educational outcomes for children from poor families and vulnerable groups. The policy effect is enjoyed by the people who need help, avoiding the “elite capture” phenomenon of the policy (33).

**Table 8:** Policy effect evaluation for boarding and non-boarding students.

Variable	Boarding students		Non-boarding students	
	(1)	(2)	(3)	(4)
	Language	Math	Language	Math
Policy effect	0.582***	0.391*	0.177	0.010
	(0.205)	(0.225)	(0.346)	(0.316)
Control variables	Yes	Yes	Yes	Yes
District fixed effects	Yes	Yes	Yes	Yes
Year fixed effects	Yes	Yes	Yes	Yes
Individual fixed effects	Yes	Yes	Yes	Yes
Observations	1,843	1,843	1,888	1,888
R-squared	0.747	0.780	0.853	0.845

***, **, and * represent significance at 1, 5, and 10% levels, respectively, and robust standard errors are in parentheses.

#### 4.6.5 Regional pilot area heterogeneity analysis

The implementation of the NIP varies at different levels. In addition to national pilot areas, provinces also have local pilot areas. This study obtains a list and implementation time of each local pilot area based on announcements related to the NIP published by each province’s education department, assessing the policy effect of the local NIP by changing the policy implementation area. We consider the short- and long-term effects of the NIP in local pilot areas. As shown in Table 9, the regression results are statistically insignificant. In evaluating the policy effects in local pilot areas, we find that the NIP has no significant impact on students’ educational outcomes. However, the effectiveness of policy implementation in these areas is significantly constrained by local fiscal capacity. To account for potential selection bias, this paper categorizes the samples into high-fiscal-surplus areas and low-fiscal-surplus areas based on the difference between local fiscal revenue and expenditure. The results also show that regardless of the level of local fiscal capacity, the policy effect in local pilot areas remains statistically insignificant.

**Table 9:** Policy effect evaluation for local NIP pilot area analysis.

Variable	Local pilot program		Local pilot program	
	(1)	(2)	(3)	(4)
	Language	Math	High school enrollment	High school enrollment
Policy effect	0.043	−0.011		
	(0.065)	(0.063)		
Policy effect 1			0.002	
			(0.035)	
Policy effect 2				0.008
				(0.008)
Control variables	Yes	Yes	Yes	Yes
District fixed effects	Yes	Yes	Yes	Yes
Year fixed effects	Yes	Yes	Yes	Yes
Individual fixed effects	Yes	Yes	Yes	Yes
Observations	6,392	6,393	3,690	3,690
R-squared	0.573	0.599	0.373	0.373

The potential reasons for this outcome may be complex. First, the National NIP has established uniform standards in pilot areas, with dedicated central government funds allocated for the construction of school canteens. These areas generally adopt the canteen meal service model, which has proven to be more effective in policy implementation. In contrast, local pilot areas, which rely primarily on funding from local governments, face more limited support. Standards vary across these local pilot areas, and in some regions, the subsidy standards for the NIP fall below the national level. The free nutritious lunch program more often adopts the enterprise meal service model, which has lower nutritional value, to save on the costs required for the construction and operation of canteens. Second, local pilot areas target less impoverished counties, which, compared with national pilot areas, have relatively better economic conditions and educational resources, and families are less sensitive to food consumption expenditure. Therefore, the NIP has limited improvement on children’s educational outcomes. Third, the effectiveness of local pilot programs is often undermined by the financial constraints of local governments and the priorities of local officials. These officials tend to allocate resources toward projects that deliver quick, visible results, such as infrastructure or economic development, rather than long-term initiatives like NIP. As a result, funding for these programs is frequently scaled back, diluting their impact and leaving rural students with fewer resources than intended. This misalignment of incentives highlights a systemic challenge in ensuring that critical social policies achieve their full potential (17).

## 5 Discussion

The NIP has effectively improved children’s educational outcomes; however, further clarification is needed concerning the mechanisms of action. This study next discusses three potential mechanisms of the NIP. First, the nutritional health mechanism analysis, second, the ability perspective mechanism, and third, the family education mechanism.

### 5.1 Nutritional health mechanism

In some rural areas, families often hold entrenched economic beliefs and lack sufficient awareness of the importance of proper nutrition. Government-led NIP, however, provided a critical external impetus for shifting these perceptions and delivering effective nutritional support. By addressing dietary gaps and promoting healthier eating habits, these initiatives create the necessary conditions for improving academic performance, ultimately helping students reach their full potential. From a nutritional health perspective, this study initially examines the impact of the NIP on rural children’s nutritional intake. The CFPS data from 2010 to 2014 collected information on the food consumed by respondents over the past week, which can proxy for children’s nutritional intake. This study constructs six binary variables for the types of food consumed by the respondents last week, encompassing meat, fish, milk, eggs, soy products, and fruit and vegetables. The results are presented in Table 10, revealing that the NIP significantly improved children’s intake of fish, milk, eggs, soy products, and fruit and vegetables, with 13.3, 5.3, 5.6, 10.1, and 13.3% increases, respectively. The intake of these nutritious foods provides children with the high-quality protein, vitamins, minerals, and dietary fiber needed for growth, enhancing their health and ensuring the nutrition required for children’s educational achievement.

**Table 10:** Mechanism analysis from a nutritional health perspective.

Variable	(1)	(2)	(3)	(4)	(5)	(6)
	Meat	Fish	Milk	Eggs	Soy Products	Fruit and Vegetables
Policy effect	−0.038	0.133***	0.053*	0.056*	0.101***	0.133***
	(0.041)	(0.034)	(0.028)	(0.030)	(0.029)	(0.050)
Control variables	Yes	Yes	Yes	Yes	Yes	Yes
District fixed effects	Yes	Yes	Yes	Yes	Yes	Yes
Year fixed effects	Yes	Yes	Yes	Yes	Yes	Yes
Individual fixed effects	Yes	Yes	Yes	Yes	Yes	Yes
Observations	4,986	4,986	4,986	4,986	4,986	4,986
R-squared	0.620	0.524	0.489	0.571	0.526	0.574

***, **, and * represent significance at 1, 5, and 10% levels, respectively, and robust standard errors are in parentheses.

### 5.2 Ability mechanism

We examine the impact of NIP on children’s cognitive and noncognitive capabilities from the ability perspective. Educational outcomes reflect children’s comprehensive capabilities, and improvements in educational outcomes are inseparable from the enhancement of children’s cognitive and noncognitive abilities. We measure cognitive abilities by referencing memory and number series test scores. While the CFPS questionnaire from 2010 to 2020 includes memory and number series test scores, different questionnaires are used in adjacent years. To facilitate comparison, we standardize memory and number series test scores from each year, with higher values indicating stronger cognitive abilities. Noncognitive abilities are considered to have long-term plasticity and significantly impact children’s educational outcomes. Therefore, we measure noncognitive abilities referencing the children’s effort, self-discipline, and concentration. The parental questionnaire asks for opinions on the following statements. “This child studies very hard,” “This child checks their homework several times after completing it to see if it is correct,” and “This child pays attention when doing things.” We code the responses from 1 to 5, with higher values indicating greater effort or better concentration. The results in Table 11, columns (1) and (2), demonstrate that the NIP significantly improved rural children’s memory test scores but had no significant impact on number series test scores. The NIP significantly improved children’s effort, concentration, and persistence. The results suggest that the NIP has partially improved rural children’s cognitive and noncognitive capabilities, enhancing children’s educational performance.

**Table 11:** Mechanism analysis from the ability perspective.

Variable	Cognitive ability		Noncognitive ability		
	(1)	(2)	(3)	(4)	(5)
	Memory	Number series	Degree of effort	Self-discipline	Concentration
Policy effect	0.233***	−0.022	0.177***	0.171**	0.096*
	(0.080)	(0.079)	(0.063)	(0.083)	(0.052)
Control variables	Yes	Yes	Yes	Yes	Yes
District fixed effects	Yes	Yes	Yes	Yes	Yes
Year fixed effects	Yes	Yes	Yes	Yes	Yes
Individual fixed effects	Yes	Yes	Yes	Yes	Yes
Observations	4,759	4,597	6,044	6,045	6,045
R-squared	0.198	0.259	0.503	0.397	0.444

***, **, and * represent significance at 1, 5, and 10% levels, respectively, and robust standard errors are in parentheses.

### 5.3 Family expectations mechanism

Individuals from lower socioeconomic backgrounds often tend to underestimate the returns on education and the likelihood of obtaining higher education, mistakenly lowering their educational aspirations. However, NIP can help shift these low expectations regarding access to higher education. According to the self-motivation model, personal expectations directly influence academic performance (34). NIP has been shown to significantly boost parents’ expectations for their children’s education. By enhancing students’ expectations for educational achievement and encouraging them to pursue higher levels of education, NIP contributes to the accumulation of human capital, fostering long-term social and economic benefits. Considering the family education mechanism, we focus on parents’ expectations for their children’s grades in the next semester, parents’ expectations for their children’s future education, and whether children attend tutoring classes. The regression results are presented in Table 12, revealing that the NIP increased parents’ educational expectations. After NIP implementation, parents’ expectations for their children’s grades increased by 2.371, and their expectations for the children’s education levels rose by 0.370. Second, the NIP altered family consumption practices, and the probability of children participating in educational training increased by 0.170. This indicates that the NIP led families to adjust their consumption structure and increase family investment in children’s education, with a “crowding in” effect on children’s education.

**Table 12:** Mechanism analysis from the family expectations perspective.

Variable	(1)	(2)	(3)
	Expected academic performance	Expected education level	Educational training
Policy effect	2.371***	0.370***	0.170*
	(0.850)	(0.112)	(0.101)
Control variables	Yes	Yes	Yes
District fixed effects	Yes	Yes	Yes
Year fixed effects	Yes	Yes	Yes
Individual fixed effects	Yes	Yes	Yes
Observations	7,014	5,408	3,849
R-squared	0.085	0.191	0.728

***, **, and * represent significance at 1% and 10% levels, respectively, and robust standard errors are in parentheses.

We further incorporate a mediation effect model that introduces family expectations and cognitive ability as explanatory variables into the baseline regression model to explore how changes in family expectations and cognitive ability jointly influence educational outcomes. The regression results are shown in Table 13. As shown in the table, the coefficients for memory cognitive ability, number series cognitive ability, and parents’ expectations for their children’s education level are all positive and statistically significant at the 1% level. However, the significance of the political effect disappears. This implies that the inclusion of family expectations and cognitive ability explains the pathway through which the NIP affects educational outcomes. The NIP affects educational outcomes by raising family expectations and improving cognitive ability.

**Table 13:** Mechanism analysis from the perspective of the joint effect.

Variable	(1)	(2)
	Expected academic performance	Expected education level
Memory cognitive ability	0.139***	0.252***
	(0.018)	(0.022)
Number series cognitive ability	0.121***	0.050**
	(0.019)	(0.021)
Expected education level	0.121***	0.162***
	(0.019)	(0.021)
Policy effect	0.172	0.076
	(0.132)	(0.123)
Control variables	Yes	Yes
District fixed effects	Yes	Yes
Year fixed effects	Yes	Yes
Individual fixed effects	Yes	Yes
Observations	3,432	3,432
R-squared	0.208	0.231

***represents significance at 1% level, and robust standard errors are in parentheses.

In terms of the magnitude of the coefficients, memory cognitive ability, number series cognitive ability, and parents’ expectations for their children’s education level have similar effects on language grades. However, in the case of math grades, number series cognitive ability has the strongest effect, followed by parental expectations of their children’s education level. This suggests that the impact of cognitive ability and parental expectations on educational outcomes varies between subjects.

## 6 Conclusion

This study employs the DID method to measure the impact of free school meals on rural children’s educational outcomes based on a quasi-natural experiment of NIP implementation. The estimation reveals that: (1) The NIP improved rural children’s math and language grades, with math grades increasing by 0.223 standard deviations and language scores increasing by 0.172 standard deviations. The NIP also increased the pass rates for language and math by 5.7 and 6.8%, respectively, and raised the high school enrolment rate by 8.2%. We also measure the effectiveness of NIP policy funds, revealing that a one yuan increase in the per capita standard of free school meals increased rural children’s math grades by 0.062 standard deviations, and language grades by 0.058 standard deviations. Dynamic effect analysis indicates that longer NIP implementation has greater improvement effects on rural children’s educational outcomes. (2) This study also conducts a series of heterogeneity analyses. The analysis of national and local pilots demonstrates that national pilot areas improved educational outcomes more significantly than local pilot areas. The analysis of different family backgrounds indicates that the NIP effectively improved the educational outcomes of children from relatively disadvantaged families. The individual-level analysis reveals that the NIP improved educational outcomes for girls more than boys, and it had a superior effect on younger children than older children. (3) Further mechanism analysis demonstrates that the NIP improved children’s educational outcomes by improving children’s nutritional health, enhancing their cognitive and noncognitive abilities, and increasing family educational expectations and educational practices.

Our findings offer insights with profound policy implications: Firstly, the NIP has shown notable success in boosting educational outcomes for vulnerable groups, such as young children, those in boarding schools, and children from impoverished families. This indicates that the NIP can enhance social mobility by improving children’s nutrition, thus accumulating human capital and increasing the likelihood of upward social mobility for the next generation. Consequently, we recommend expanding the coverage of NIP to ensure that all children in rural compulsory education can benefit from these initiatives. Secondly, we advocate for the implementation of age-based differentiated subsidy policies, providing targeted financial support according to the nutritional needs of children of different age groups, and devising corresponding differentiated subsidy plans. We also recommend broadening the age range of nutritional subsidies to include early childhood, recognizing that early interventions in human capital development offer high returns on relatively small investments, a principle particularly relevant in the early years. Lastly, we emphasize the importance of strengthening oversight to enhance the efficiency of fund usage and ensure that the funds directly benefit children. While the national NIP is relatively well-regulated, local regulatory efforts are lacking, which may be one of the reasons for the suboptimal performance of the local NIP. We urge the acceleration of strengthening regulatory mechanisms for NIP to guarantee their efficacy from a systemic perspective.

## 7 Limitation

While this paper provides an in-depth examination of the impact of the NIP on educational outcomes, important issues still deserve attention in future research. In the future, as the NIP continues and more data becomes available (such as specific nutritional data and physical development status), research can be expanded to include the impact of the NIP on the higher education and long-term labour market performance of rural children for a more comprehensive assessment.

## Data Availability

The datasets presented in this study can be found in online repositories. The names of the repository/repositories and accession number(s) can be found in the article/supplementary material.
